# Real Fakes: The Epistemology of Online Misinformation

**DOI:** 10.1007/s13347-022-00581-9

**Published:** 2022-08-31

**Authors:** Keith Raymond Harris

**Affiliations:** Ruhr-Universität Bochum, Bochum, Germany

**Keywords:** Deepfakes, Epistemology, Fake news, Social epistemology, Social media, Warrant

## Abstract

Many of our beliefs are acquired online. Online epistemic environments are replete with fake news, fake science, fake photographs and videos, and fake people in the form of trolls and social bots. The purpose of this paper is to investigate the threat that such online fakes pose to the acquisition of knowledge. I argue that fakes can interfere with one or more of the truth, belief, and warrant conditions on knowledge. I devote most of my attention to the effects of online fakes on satisfaction of the warrant condition, as these have received comparatively little attention. I consider three accounts of the conditions under which fakes compromise the warrant condition. I argue for the third of these accounts, according to which the propensity of fakes to exist in an environment threatens warrant acquisition in that environment. Finally, I consider some limitations on the epistemic threat of fakes and suggest some strategies by which this threat can be mitigated.

## Introduction

It is sometimes suggested that the modern world is uniquely epistemically dysfunctional. The current epistemic dysfunction is evident in the proliferation and social influence of fake news, outlandish conspiracy theories, and related phenomena, and is often attributed to the internet. This causal explanation is no doubt overly simple, as fake news (Novaes & de Ridder, 2021; Pepp et al., 2019), and conspiracy theories (Butter, 2020; Pagán, 2008, 2020; Teter, 2020; Uscinski & Parent, 2014; Yablokov, 2020) are hardly novel phenomena. Moreover, one need not look far back into the history of the mainstream press to find an institution whose outputs were systemically distorted by racism, sexism, and other prejudices (González & Torres, 2011: ch. 1).

Still, there is something new about the contemporary form of epistemic dysfunction. While misinformation has a long history, the expanded role of the internet in epistemic life gives rise to a relatively new challenge. Even as the internet often provides access to accurate information and reliable sources, it often simultaneously immerses these in a sea of counterfeits. Authentic news may be obscured by fake news, authentic scientific findings by fake science, genuine photographs and videos by doctored photographs and deepfakes, real people by trolls and bots, and so on. In each case, authentic forms and sources of information may be difficult to distinguish from their fake counterparts.

The purpose of this paper is to consider the effects of online fakes on knowledge and, in particular, the pathways by which fakes interfere with knowledge acquisition and the conditions under which they do so. After providing a brief overview of various kinds of fakes, I turn to the pathways by which fakes might interfere with the acquisition of knowledge. I begin with the effects of fakes on the satisfaction of the truth and belief conditions on knowledge. Then, I examine the effect of fakes on satisfaction of the warrant condition on knowledge. I devote considerable attention to this latter issue, as it has received little attention and is of greater epistemological complexity than the effects of fakes on the truth and belief conditions. The epistemic threat of fakes is especially pernicious, I ultimately argue, because it does not depend on the existence of fakes in an environment. Instead, the mere probable existence of fakes in an environment is enough to threaten knowledge.

## Varieties of Fakes

Epistemologists have long been preoccupied with the fake. Descartes famously sought the limits of what could be faked (1641/1995). Epistemologists writing more recently have modernized Descartes’s skeptical possibilities to recognize the potential for technologies to be used in the generation of fakes (Putnam, 1981; Vogel, 1990). Barn facades and other fakes have been used to challenge proposed analyses of knowledge (Ginet, 1988; Goldman, 1979). In many cases, the fakes discussed by philosophers are bare possibilities, rather than features of everyday life. Here, I am concerned with the epistemic effects of kinds of fakes that are, or plausibly will be, routinely encountered online. The focus on online fakes is not meant to imply that fakes only exist online. Rather, focus on online fakes is intended to acknowledge the reality that much of the information individuals in the modern world encounter is, for better or worse, encountered online. In this section, I provide an overview of the kinds of fakes that are, or will be, regularly encountered online. Then, in the remaining sections of the paper, I aim to show how these diverse types of fakes compromise the attainment of warrant in similar ways.

Especially since the Brexit referendum and the election of Donald Trump to the US presidency in 2016, epistemologists and other scholars have devoted a good deal of attention to fake news (Bernecker et al., 2021; Gelfert, 2018; Grundmann, 2020; Jaster & Lanius, 2018; Levy, 2017). Much of the philosophical work in this area has been devoted to defining fake news. This has proven to be a difficult project in part because the generation of fake news is apparently driven by divergent motives and in part because the term “fake news” is used to refer to a wide range of phenomena (Habgood-Coote, 2019). Beginning with the first point, the otherwise plausible association of fake news with deceptive intent faces the challenge that some apparently paradigmatic instances of fake news have been created, not to deceive, but for the purpose of generating web traffic and, in turn, ad revenue (Hughes & Waismel-Manor, 2020). It has been argued that the possibility of principally profit-driven fake news is consistent with the definition of fake news as intended to deceive, insofar as deception helps to maximize web traffic (Rini, 2017). However, it seems at least conceivable that there could be fake new with no deceptive intent. As to the second point, the term “fake news” has been used to refer to phenomena ranging from satire, to political propaganda, to clickbait, and to a wide range of content and institutions that are critical of rightwing populist political figures in the USA.^1^

Fortunately, it is enough for present purposes to stipulate a definition of “fake news”. For present purposes, we are interested in counterparts of legitimate news that represent themselves as genuine, but are produced without the guiding aim of accuracy.^2^ Instead, fake news is produced for political or financial purposes, and without even a pragmatic adherence to truth to better pursue these purposes. Herein lies the distinction with genuine news. It is typical for genuine news outlets to aim, in some sense, at the pursuit of profit. However, this pursuit is largely, albeit imperfectly, constrained by the aim of accuracy, if only because departures from this aim undermine the long-term pursuit of profit. As a consequence, genuine news outlets employ practices like professionalization, fact-checking, and editorial oversight.^3^ Under this definition, it is in principle possible for fake news stories to be true (cf. Fallis & Mathiesen, 2019: 11–12). However, because the creators of fake news do not aim at truth, this will occur accidentally, if at all. Given this definition, I will not use “fake news” to refer to satirical news stories or to stories or institutions of the kind unwarrantedly targeted by rightwing populist figures. To the extent that phenomena belonging to these latter categories are false and difficult to distinguish from their authentic counterparts, the analysis to follow will apply to some members of these categories. However, we need not focus on such atypical instances here.

Just as news has a fake counterpart, so too does science. Following Emmanuel Genot and Erik J. Olsson (2021), I will understand fake science as fraudulent science—scientific publications that misrepresent the empirical evidence or its import. Thus, paradigmatic instances of fake science include articles in which either the methodology of the study or the conclusions of the study are represented inaccurately. Notably, fake science is relatively prominent on the internet. For example, as Genot and Olsson (2021) report, findings based on scientific fraud are typically more readily accessible than retractions of such findings. Moreover, fraudulent findings typically receive more media attention than retractions. Consequently, a casual inquiry into some scientific topic will likely turn up the fraudulent finding, even when it has been retracted. 

 While the categories of fakes discussed thus far are already regularly encountered in the online environment, deepfakes remain, as of this writing, comparatively rare. The term “deepfake” is most commonly used to refer to videos generated through deep learning processes that allow for an individual’s likeness to be superimposed onto a figure in an existing video. Deepfakes thus do for video footage something like what Photoshop did for photographs. These technologies grant users the power to generate manipulated content with relative ease. In doing so, these technologies bring about similar epistemic effects. It has previously been argued that Photoshop undermines the power of photographic evidence (Cavedon-Taylor, 2013). More recently, it has been argued that deepfakes undermine the power of video evidence (Carlson, 2021; Fallis, 2020; Kerner & Risse, 2021; Rini, 2020). Scholars fear that, just as doctored photographs are now routinely encountered online, misleading deepfakes will soon likewise be commonplace.

Trolls and social bots make up the final category of fakes to be considered here. Trolls, also known as “sockpuppets”, are real humans that post insincerely online, often under fabricated identities. In recent years, online trolling has been weaponized to the extent that state actors now push disinformation via organized systems of trolls—sometimes called “troll farms” or “factories” (Linvell & Warren, 2020; Zannetou et al., 2019). While troll accounts are directly operated by real people, those that misrepresent their identities can be understood as fake people (Rini, 2021: 41). I focus on trolls of this kind here. Social bots operate on social media platforms and enjoy some degree of autonomy from their human programmers. Despite this degree of autonomy, social bots can be deployed for various purposes, including advertising and political propagandizing. Social bots often represent themselves as human users, with fake biographical details inserted in their profiles (Ferrara et al., 2016). For this reason, many social bots can sensibly be regarded as fake people. In addition to bots and trolls, there are cyborgs—accounts whose behavior is partly the product of direct human action and partly the product of automated activity (Chu et al., 2012). Cyborgs combine features of trolls and bots and may likewise be classified as fake people. Because cyborgs inherit their characteristics from the properties of trolls and bots, I do not discuss cyborgs independently of these other entities below. Fake people, in the forms of trolls and bots, are interconnected with some of the other categories of fakes discussed thus far. Trolls and social bots have been implicated in the spread of fake news, conspiracy theories, and the like (Bastos & Mercea, 2019; Broniatowski et al., 2018; Howard & Kollanyi, 2016; Samuels, 2018; Shorey & Howard, 2016; Swaine, 2018).

This overview of fakes is intended to briefly illustrate some of the epistemic challenges facing internet users. In short, genuine items and sources of information are often now and will likely increasingly be obscured by fake counterparts. In this way, fakes give life to skeptical possibilities of the sort that have often commanded the attention of epistemologists. Naturally, distinct kinds of fakes may pose distinct epistemic challenges. For example, fake science and deepfakes, but not fake news reports, may accidentally be published in otherwise credible outlets.^4^ Moreover, science, and especially certain subfields, can plausibly be given an anti-realist interpretation and thus faces fundamental issues of truth that are less pressing in other domains. In light of such differences, different kinds of fakes plausibly have different kinds of effects on the credibility of existing institutions. However, in this paper, my primary focus is on the shared epistemic consequences of these distinct kinds of fakes. The present paper may thus be understood as groundwork for future inquiry into the unique epistemic consequences of distinct kinds of fakes.

## Epistemic Threats

Beginning in Section 3, I will discuss some general ways in which fakes pose epistemic threats. To simplify this discussion, let us define an epistemic threat as a threat to the acquisition or retention of knowledge. For some purposes, it might be useful to embrace a more expansive conception of epistemic threat—for instance one that would encompass threats to understanding. However, to focus the present discussion, it will be useful to focus specifically on how fakes threaten knowledge. One advantage of this focus is that, compared to the state of understanding, there is a greater degree of agreement among epistemologists as to what knowledge requires. Moreover, if understanding requires knowledge, as some epistemologists contend (Grimm, 2006; Kelp, 2018), any epistemic threat to knowledge will likewise be a threat to understanding. Finally, the analysis of knowledge in terms of a set of necessary and sufficient conditions allows for a simple breakdown of some pathways by which fakes might threaten knowledge.

I will assume that knowledge is warranted true belief. That knowledge at least requires true belief is largely uncontroversial. How to understand warrant—that is, whatever fills the gap between true belief and knowledge—is the subject of ongoing dispute. It is widely thought that warrant is a matter of justification and the absence of epistemic luck. While some epistemologists explicitly deny the necessity of justification for knowledge, this denial is typically premised on a narrow, internalist conception of justification (Goldman, 1976), which we need not adopt. Debate persists among epistemologists as to how best to capture the incompatibility of knowledge with luck (Greco, 2010; Pritchard, 2009). Rather than wading into these debates, I will understand warrant as whatever makes the difference between true belief and knowledge, without committing to any detailed proposals as to its nature.

## Fakes and Truth

Let us begin with what is perhaps the most straightforward way in which fakes might interfere with knowledge. Some fakes may be deceptive, in the sense that they lead to the formation of false beliefs. For this to occur, the fake must be encountered by the subject, either directly or through an intermediary from whom the subject receives information, thereby causing a modification in that subject’s doxastic states. Fakes of all kinds can be deceptive. Reading a fake news story might lead one to form or abandon the belief that a given politician is corrupt. Encountering fake science might lead one to believe that Ivermectin prevents COVID-19. Encountering a deepfake might lead one to believe that a celebrity uttered a slur. And so on.

Insofar as fake people disseminate other forms of fakes, they may drive the straightforward form of deception highlighted above. They may do so not only by spreading fakes, but by offering misleading higher-order evidence as to the legitimacy of these fakes.^5^ Additionally, trolls and social bots add a further layer of deception beyond that posed by the other categories of fakes. Trolls and social bots may be deceptive not only with respect to the content they share, but also with respect to their very identities. In this latter way, trolls and social bots might also be deceptive with respect to what real human persons are like.

To conclude this section, let us consider a less direct way in which fakes may contribute to deception. As Regina Rini (2020) argues, the real possibility that any particular video is a deepfake may reduce the costs of other forms of deception. For example, one reason why a politician might hesitate to lie is the concern that video footage, either of the lie itself or of the sort that would prove the lie to be false, would make the lie politically damaging. However, if video footage can be credibly written off as a deepfake, then the chances of one’s lies being exposed as such by video footage are reduced. In this way, deepfakes reduce the incentive not to lie (Chesney & Citron, 2019). The point generalizes to other kinds of fakes. For example, when apparent proof that one engaged in deception can be written off as “fake news” one loses some incentive not to lie. Fakes thus reduce the costs of deception, and thereby may contribute in an indirect way to deception by other means.

## Fakes and Belief

The recognition that the online environment is lousy with fake news, fake science, fake audiovisual content, and even fake people might cause confusion and, ultimately, reluctance to form beliefs based on content encountered online. Indeed, this is precisely the result that some disinformation is intended to produce. For example, the so-called firehose-of-falsehood model of disinformation, typically identified with Russian propaganda (Paul & Matthews, 2016), operates by introducing and amplifying incompatible narratives in the epistemic environment (Pomerantsev, 2014). Notably, trolls are sometimes integral to this strategy (Broniatowski et al., 2018). This model has been adopted by domestic political actors in the US context, and the strategy has been memorably described by former Donald Trump advisor Steve Bannon in terms of “flood[ing] the zone with shit” (Stengel, 2020). In an environment so flooded, internet users may succumb to a state of disorientation (Benkler et al., 2018), in which they hesitate to believe anything at all (Pomerantsev, 2019; Rini, 2021).

To recognize that fakes might lead to the reluctance to form beliefs, we need not assume that belief—or in this case non-belief—is under voluntary control. Recognition of the problem of fakes might produce changes in how a subject automatically updates beliefs in light of news stories, videos, and so on. But fakes may also result in a more deliberate, albeit indirect, reluctance to form beliefs. Sven Bernecker (2021), for example, advocates a form of “news abstinence”—the reduction of news consumption and the avoidance of news from some sources and concerning certain topics—as a response to the problem of fake news. This strategy, and indeed any hesitation to form beliefs based on online content, comes at a cost. Knowledge requires belief and, consequently, hesitation to form beliefs limits one’s opportunities for knowledge. In this way, the attempt to avoid fakes may compromise one’s epistemic prospects.

The threat of fakes may discourage the attainment of knowledge in subtler ways. Individuals may be discouraged from activities that would lead to the formation of true beliefs not because they fear that deception is unavoidable, but instead because they recognize that, in an information environment populated by fakes, reliable inquiry is likely to be time-consuming. For instance, even one who is confident that there are means to distinguish between real and fake science may regard the work that would be required to do so as unacceptably costly. In this way, awareness of the threat of fakes may subtly discourage would-be knowers.

So far in this section, I have focused on how fakes might discourage individuals from forming beliefs based on content they encounter online. This effect may reverberate more widely if individuals come to recognize that, as discussed in Section 3, fakes reduce the incentive not to engage in deception. One familiar with this point might, for instance, view all testimony with heightened suspicion.

## Fakes and Warrant

In the preceding two sections, I discussed how fakes may promote false belief and suppress true belief. These are the most straightforward effects of fakes and, insofar as it is the psychological rather than the normative dimensions of knowledge that influences behavior, arguably the most practically significant. There are further ways in which fakes threaten knowledge that have received comparatively little attention from non-philosophers but are especially interesting from an epistemological perspective. In the remainder of the paper, I consider in detail how and under what circumstances fakes undermine warrant and thus how, even where fakes do not interfere with true belief, they nonetheless interfere with knowledge. I begin, in this section, by considering some mechanisms by which fakes might interfere with warrant. As I noted above, I do not wish here to make controversial epistemological assumptions concerning the nature of warrant. For this reason, I present in this section a series of not necessarily incompatible proposals as to how fakes might interfere with knowledge without committing to any particular proposal(s). In the process, we will see how various epistemological approaches can be applied to the issue of online fakes. Then, in Sections 6 and 7, I discuss the conditions under which fakes threaten knowledge. 

Let us begin with Don Fallis’s (2020) recent proposal as to how deepfakes interfere with knowledge. As we will see, the proposal can be generalized to describe how other kinds of fakes interfere with knowledge. The core of Fallis’s proposal is that deepfakes reduce the amount of information carried by video footage. Fallis’s proposal is perhaps best illustrated by his analogy with Batesian mimicry in non-human animals. Batesian mimic species are species that are no danger to predator species, but that mimic signals associated with species that are dangerous—usually in the sense of being venomous or poisonous—to predator species. Fallis’s preferred example of a Batesian mimic is the king snake, some species of which have coloring that closely resembles that of the venomous coral snake. Having the coloring of a coral snake carries the information that a given snake is venomous. However, according to Fallis, the amount of information carried by this coloring is reduced in environments that also contain similarly patterned but non-venomous king snakes. This is because the amount of information conveyed by a snake’s pattern is contingent upon the relative conditional probabilities of a snake having the relevant coloring given that it is poisonous and given that it is not poisonous. Non-venomous mimics effectively reduce the reliability of the snake’s signal.

Fallis suggests that deepfakes have a comparable effect on the amount of information conveyed by videos. Ordinarily, a video depicting *p* would carry the information that *p*. However, deepfakes raise the probability that some video depicts *p* even though *p* is false. In this way, deepfakes reduce the information conveyed by video footage. Put differently, deepfakes reduce the amount of evidence that is provided by video.^6^ To better grasp this point, consider the following illustration. Suppose that, in the near future, one happens across an online video that seems to show a prominent politician committing a gaffe. In the absence of a means for generating convincing yet fake video content, such a video would plausibly provide strong evidence for the proposition that the politician committed the gaffe. However, the emergence of deepfakes and related techniques plausibly limits the amount of information conveyed by the video. Even if such a video once offered sufficient support for an audience to know that the politician committed the gaffe, deepfakes would seem to undermine the ability to acquire knowledge in this way.

Fallis’s framework for understanding the epistemic threat of deepfakes can be applied to other varieties of fakes. Peter J. Graham (2000) suggests a similar mechanism by which fraudulent news reports undermine the ability to acquire knowledge from genuine news reports.^7^ Similarly, Photoshop and related techniques plausibly reduce the information conveyed by photographs. This is because, given such technologies, the chance that a photograph exists depicting some state of affairs is relatively high, even if the state of affairs is non-actual. Likewise, the realistic possibility that any particular news report is fake news plausibly goes some way toward depriving that report of its ability to carry information. In short, fakes undermine the informational content of their real counterparts. Insofar as knowledge requires information or, equivalently, evidence, fakes thereby interfere with the acquisition and retention of knowledge.

The epistemic ill-effects of fakes can be captured in other terms. Epistemologists have often suggested that knowledge requires the ability to rule out certain relevant alternatives. For example, Barry Stroud (1984: ch. 1) suggests that, to know that the bird in one’s backyard is a goldfinch, one must under some circumstances be able to rule out the alternative that it is a canary. In this case, the possibility that must be eliminated is one that is incompatible with the truth of the proposition potentially known. However, as Stroud adds, knowledge that some proposition is true sometimes requires that certain possibilities, not incompatible with the target proposition, be ruled out. Consider an example. If one has taken a hallucinogenic drug and subsequently seems to see that one’s bed is covered in leaves, one must rule out the possibility that the leaves are the product of hallucination to know that the bed is covered in leaves (Stroud, 1984). This is even though it is consistent with one’s hallucinating the presence of leaves that one’s bed is actually covered in leaves.

Notably, in Stroud’s hallucinogenic drug example, Stroud stipulates that one has actually taken the hallucinogenic drug. It is partly for this reason that the possibility that one is hallucinating leaves is a *relevant* alternative. While epistemologists differ in what they are willing to concede to the skeptic, one would, at a minimum, meet more resistance if one were to suggest that perceptual knowledge *always* requires that one rule out the possibility that one’s perceptions are due to hallucination. Given that one has taken a hallucinogenic drug, it is a relevant alternative that one’s perceptions are due to hallucination, even if this is not typically a relevant alternative.

Christopher Blake-Turner (2020) argues that fake news threatens knowledge by introducing relevant alternatives. When one comes to believe that *p* based on a news report that *p*, it may be a relevant alternative that the news report is a fake. Like the possibility that one is hallucinating leaves, the possibility that a news report is fake is consistent with the truth of the target proposition. Fake news threatens the acquisition of knowledge from news reports insofar as the possibility that a given report is fake news is a relevant alternative. Notably, this possibility is a realistic one, sometimes instantiated in the real world. In philosophical jargon, there are nearby possible worlds in which the news stories on which one bases one’s beliefs are fake.

Blake-Turner’s point concerning the epistemic effects of fake news generalizes. Matthew Carlson (2021) argues that deepfakes introduce relevant alternatives for many of our beliefs. Similarly, that one’s sources are fake science, Photoshopped photographs, or some other form of fake, are, at least in some cases, relevant alternatives. The epistemic ill-effects of fakes may thus be understood in terms of the introduction of relevant alternatives.

Finally, let us consider how the epistemic threat of fakes might be understood according to externalist epistemologies. Fallis writes the following about a potential consequence of deepfakes:

[E]ven after watching a genuine video and acquiring true beliefs, one might not end up with knowledge because one’s process of forming beliefs is not sufficiently reliable (2020).

Fallis’s suggestion is that deepfakes might compromise the reliability of certain belief-forming methods—namely those based on the viewing of video footage. Given a reliabilist approach to knowledge, the further consequence is that deepfakes might interfere with the acquisition of knowledge. As with the other threats to knowledge considered above, Fallis’s point can be extended to other kinds of fakes. Fake news compromises the reliability of forming beliefs based on news reports. Fake science compromises the reliability of forming beliefs based on scientific publications. And so on.

Beyond the process reliabilist approach, alternative externalisms can capture the epistemic effects of fakes in modal terms. Some epistemologists take knowledge to require either sensitivity (Nozick, 1981) or safety (Sosa, 1999). A belief is sensitive just in case, if its contents were false, the subject would not believe it. A belief is safe just in case, if the subject were to believe it, it would not be false. As with the relevant alternatives approach, whether the safety and sensitivity conditions are satisfied can be understood in terms of what is true in nearby possible worlds in which a belief is formed by the same method.^8^ Fakes interfere with the satisfaction of both conditions. Let us suppose that the proposition *p* is the proposition that some prominent politician, *s*, committed an embarrassing gaffe. Suppose this proposition is true in the actual world, and that one comes to believe the proposition based on authentic video footage that shows *s* committing the gaffe. While the belief is true, fakes might render this belief both insensitive and unsafe, and hence the belief might fail to constitute knowledge. Let us begin with sensitivity. When we consider the nearest possible worlds in which *p* is false, these worlds may well contain a deepfake showing *s* committing the gaffe. The belief is thus plausibly insensitive to the truth of its contents. Likewise, when we consider nearby possible worlds in which the subject believes *p*¸ some of these worlds are plausibly such that *p* is false. These are worlds in which, despite the falsity of *p*, there exists a deepfake that convinces the subject that *p*. The belief is thus plausibly unsafe. More generally, fakes arguably compromise the sensitivity and safety of beliefs by changing what would be true in certain possible worlds.

I do not pretend to have made a decisive case that fakes render various beliefs insensitive or unsafe. Epistemologists have struggled to assess the nearness of possible worlds and to determine precisely which possible worlds are relevant to the satisfaction of the modal conditions (Baumann, 2008, 2016: ch. 2; Bogardus, 2014; Comesaña, 2005; Craig, 1990: ch. 3). For present purposes, my intention has been to state in general terms how fakes might threaten knowledge by compromising warrant. Interfering with the sensitivity or safety of beliefs formed using certain methods is one way in which this might plausibly occur. Epistemologists are likely to disagree concerning the mechanism by which fakes threaten knowledge. However, regardless of which approach one favors—whether it is among those discussed here or some further alternative—a question remains concerning the conditions under which fakes threaten knowledge. I turn to this issue in the next section.

## Two Accounts of the Epistemic Threat of Fakes

I have argued that fakes can threaten knowledge by interfering with one or more of the truth, belief, and warrant conditions on knowledge. When precisely fakes interfere with satisfaction of each of the first two conditions is a relatively straightforward matter. A given fake can only directly compromise the truth of one’s beliefs by being encountered and thereby causing the subject to abandon a true belief or to form a false belief. As we have seen, fakes might less directly impact the truth of a subject’s beliefs by removing a disincentive against deception or by influencing intermediaries from whom the subject receives information. Fakes may also have a straightforward effect on the belief condition. Excessive skepticism attributable to awareness of the problem of fakes may prevent one from forming true beliefs based on authentic information. But the conditions under which fakes compromise the warrant condition are less straightforward. To illustrate, let us start by considering two possibilities.

One possibility is that it is the real existence of fakes that compromises the warrant condition. Call this the *existence view*. This is the possibility most naturally suggested by Fallis’s (2020) analogy between deepfakes and Batesian mimics. The amount of information conveyed by a coral snake’s coloring is plausibly contingent upon the number of mimics in that environment. Likewise, one might think, the amount of information conveyed by genuine news, photographs, videos, and the like is contingent upon the number of counterpart fakes in the environment. For example, the amount of information conveyed by video footage is inversely proportional to the number of deepfakes in an environment. Similarly, the amount of information conveyed by news reports is inversely proportional to the number of fake news reports in the environment. And so on.

The existence view requires some clarification, as the effects of fakes on warrant plausibly depends on the relevance of the fakes in question. Consider that the vast majority of extant deepfakes are pornographic (Ajder et al., 2019; Cox, 2019). While such deepfakes create a host of moral concerns (Harris, 2021; Öhman, 2020; Paris & Donovan, 2019; Young, 2021: ch. 11), there is reason to think that the epistemic ill-effects of such deepfakes are limited. Similarly to how the presence of fake barns in a region intuitively would not compromise the warrant for beliefs concerning the existence of tractors and cows in that region, it seems that the enormous quantity of pornographic deepfakes that exist online do not undermine the warrant that can be gleaned from viewing a video of a prominent politician delivering a speech. An analogy with the epistemology of testimony further suggests the significance of relevance. One might think that the prevalence of lies in the context of a used car dealership undermines the acquisition of warrant concerning the quality of the cars on the lot, without allowing that the prevalence of such lies undermines the acquisition of warrant for beliefs about entirely unconnected matters. None of this is to say that pornographic deepfakes will have no epistemic effects. First, the observation of extent deepfakes, and awareness of their increasing quality, may lead one to be skeptical about video evidence and thus reluctant to form beliefs based on such evidence. Second, pornographic deepfakes may well compromise the warrant one can obtain from videos whose content is similar in some relevant respect to that of pornographic deepfakes.

The basic point that the epistemic ill-effects of fakes depend on their content generalizes. Plausibly, deepfakes of political figures and celebrities do not undermine the warrant that can be obtained from viewing videos of one’s non-famous friends, for example. Thus, on the most plausible version of the existence view, the warrant to be obtained from any given video footage is contingent only on the existence of deepfakes whose content is in some way relevant. This point can be restated in terms of various views discussed in Section 5. For example, one might say that deepfakes with content of a certain kind are analogous to mimics of particular species. Just as the existence of a snake whose patterning mimics that of another snake species would not undermine the informational content of all animal patterning, the existence of a deepfake showing a politician committing a gaffe does not undermine the informational content of all videos. Likewise, the process of forming beliefs based on videos of friends or family may be reliable even if forming beliefs based on videos of political figures committing gaffes is not. While I have focused here principally on deepfakes, similar points hold true of other varieties of fakes. The existence of a fake news report or fake photograph in the domain of sports does not compromise the warrant to be gained from news reports and photographs in all other domains, for instance.

In summary, the most plausible version of the existence view will have it that the epistemic ill-effects of fakes are restricted according to a principle of relevance. Determining a nonarbitrary principle of relevance is a difficult task, and one I will not attempt to resolve here. As the above point about deepfakes of politicians committing gaffes not undermining the attainment of warrant from videos of one’s friends illustrates, individual cases elicit intuitions that are of some guidance here. However, it is consistent with the aforementioned intuitive judgment that the principle of relevance binds together content involving particular individuals, concerning particular domains like politics and sports, and further alternatives. This sort of difficulty is not specific to the epistemology of online fakes.^9^ In cases of animal species, for example, it is plausible that mimic species may undermine warrant for beliefs other than those concerning members of the species they mimic.^10^ Even if a king snake’s patterning does not compromise the informational content of all animal patterns, it might compromise the informational content of the patterns of snakes other than coral snakes, for example. I flag this problem and its ubiquity here, in part, to show the continuity between the epistemology of online fakes and more traditional epistemological problems.

I have thus far argued that the most plausible version of the existence view would take the epistemic threat of fakes to be limited according to a principle of relevance, even if this principle is difficult to state. Any plausible account of the epistemic threat of fakes must recognize a second restriction. At the time of this writing, deepfakes remain relatively unconvincing. Even if deepfake technology continues to advance, and produces increasingly convincing results, it remains the case that many deepfakes that have been produced and will be produced in the immediate future are easily distinguishable from authentic videos. Similarly, doctored photographs can vary dramatically in their quality. Text-based fakes—in the form of fake news reports or fake science—also exhibit varying degrees of convincingness. There is little reason to suppose that highly unconvincing fakes will compromise the warrant to be obtained from their authentic counterparts. The epistemic ill-effects of fakes are thus limited to convincing fakes. Of course, a given fake may be convincing to one audience and not to another. For this reason, the epistemic threat of fakes is relativized to particular audiences. This point can again be elucidated by appeal to examples from Section 5. A king snake’s patterning may reduce the informational content of a coral snake’s patterning relative to a novice, but not to an ophiologist.^11^ Similarly, the ability to distinguish between goldfinches and canaries may enable an ornithologist, but not a layman, to know the species of a particular bird. As in these cases, a heightened ability to distinguish between fakes and their authentic counterparts may limit the epistemic ill-effects of fakes. I develop this point further in Section 8.

Let us restate the existence view now that we have clarified some of its key aspects. According to this view, it is the existence of fakes that are both relevant and convincing that interfere with the attainment of warrant from their authentic counterparts. Let us consider an alternative.

According to what I will call the *ability view*, it is the existence of an ability to introduce relevant and convincing fakes into an environment, rather than the existence of fakes themselves in that environment, that interferes with the acquisition of warrant. The ability view takes its basic motivation from the notion that, whether or not there *are* fakes in a given environment, it is of epistemic significance that there *could be*. Something like the ability view underlies many classical skeptical arguments. For Descartes, it is the fact that a powerful, malevolent deceiver *could* create a convincing illusion that threatens much of his knowledge.^12^ The ability to produce convincing fakes also appears epistemically significant in less outlandish cases. Consider the case of counterfeit currency.^13^ If a given currency were especially easy to counterfeit, then the mere appearance of that currency would provide limited warrant for its genuineness, even if as a matter of fact few or no counterfeits exist. Let us turn to a more immediately pertinent example. Prior to emergence of deepfake technology, viewing any lifelike video of a prominent politician doing F would plausibly have been strong evidence that the politician did F. However, supposing that deepfake technology invests in individuals the ability to generate inauthentic but lifelike videos that appear to show that politician doing F, one might conclude that such a video is no longer strong evidence. Arguably, deepfake technology doesn’t yet produce sufficiently lifelike results to present a serious epistemic threat. If this is right then, according to the ability view, deepfakes will present an epistemic threat as soon as the technology is sufficiently advanced. The implications of the ability view thus differ from those of the existence view, according to which the threat would only emerge through the proliferation of deepfake videos.

There is some reason to favor the ability view over the existence view. Consider an example. Suppose that, based on authentic video footage disseminated shortly before an election, one comes to believe that *p*—a prominent politician *s* used a slur. Suppose that, as it happens, there are no convincing and relevant deepfakes in existence. As we have seen, stating just exactly what would constitute a relevant deepfake is no simple matter. One might think that relevant deepfakes would be deepfakes involving *s*, deepfakes involving people using slurs, deepfakes involving prominent politicians using slurs, or some further possibility. But let us assume that no deepfake belonging to any of these categories exists. Finally, suppose that convincing deepfakes showing *s* using slurs could easily be generated. In fact, we might suppose that the political opposition was about to create such a deepfake, but cancelled the plan when the genuine video was uncovered. In such a case, there is an important sense in which one could very easily have been duped by a deepfake into believing that *s* used a slur. Intuitively, this point is epistemically relevant, but its relevance is not captured by the existence view. More generally, the existence view seems to place too much stock on what is the case, and not enough on what could easily have been the case.

However, the ability view faces its own difficulties. One concern for the view is that it arguably has implausibly skeptical consequences. This is because the ability to generate fakes, at least of some kinds, already exists. For example, photograph editing tools are available to nearly all internet users. Of course, it is one thing to be able to make doctored photographs, and quite another to be able to create convincing doctored photographs. Still, many individuals can do the latter, and it would seem implausible to suppose that photographic evidence alone is never sufficient for knowledge. Here an analogy with the epistemology of testimony is helpful. While debate between reductionists (Adler, 1994; Fricker, 1994; Hume, 1748/1999) and non-reductionists (Coady, 1992; Goldman, 1999; Hardwig, 1985; Reid, 1983; Williamson, 2000) persists, it is all but universally agreed that one can sometimes acquire knowledge via testimony. This is despite the fact that, as a general matter, it is always *possible* for the speaker to testify falsely. By analogy, it seems plausible that one can form knowledge based on authentic content encountered online, even when a fake, convincing, and relevant counterpart *could have* been generated and encountered in its place.

A related problem for the ability view is that it seems not to account for some ways in which the epistemic threat of fakes has increased. It has long been possible to create convincing fakes. What novel technologies do is principally to make this process easier and cheaper. CGI and related techniques have long allowed for the creation of convincing fake video footage, albeit through expensive and painstaking processes. Deepfake creation tools promise to make the generation of convincing fake video footage far easier and cheaper. More generally, recent technologies tend to expand the ability to generate and disseminate fakes. But, given that the ability itself is not new, the ability view appears to misdiagnose the epistemic threat of fakes. It might be objected that, on the most plausible construal of the ability view, it is the *widespread* ability to generate and disseminate fakes, rather than the possession of that ability by someone, somewhere, that threatens warrant. This suggestion appears plausible, but only insofar as, as more individuals possess the relevant ability, it becomes increasingly likely that convincing and relevant fakes will exist in the environment. This modified ability view, then, derives its plausibility from similarity to the view defended in Section 7.

A final concern for the ability view is that it fails to account for the unequal vulnerability of knowledge according to its object. For example, compare the acquisition of knowledge that some prominent politician uttered a slur to acquisition of knowledge that a politician uttered a platitude.^14^ Such knowledge might in principle be acquired from a news report or from video footage. Intuitively, however, acquisition of the former knowledge by some such method is under greater threat from fakes than acquisition of the latter knowledge by the same method. This is even though it is equally possible to generate fakes that would bear on both objects of knowledge. The ability view does not account for this difference, and thus, the intuitive judgment in this case is a challenge for the ability view. In Section 7, turn to an alternative view that better accounts for the present intuition.

Before turning to this alternative view, it is worth noting here that I have thus far treated the ability and existence views as objective, in the sense that it is the real existence of fakes, or abilities to produce fakes, that threaten warrant. Subjective alternatives of these views, and of the view considered in Section 7, might also be imagined. For example, one might suppose that, whether or not individuals can produce convincing and relevant fakes, a subject’s belief that individuals have such an ability is enough to compromise that individual’s attainment of warrant. I focus for the sake of simplicity on objective variants of the views in question, but I do not pretend to offer decisive reason to favor objective views over subjective ones.

## The Propensity View

According to what I will call the *propensity view*, it is the propensity of convincing and relevant fakes to exist in an environment that threatens the acquisition of warrant from legitimate information in that environment. Crucially, there may be a propensity for convincing and relevant fakes to exist in an environment even if they do not actually exist. The relevant propensity depends, in part, on the ability and motivation of individuals to insert fakes into the environment and, in part, on the ability and motivation of individuals to remove fakes from the environment. However, it would be a mistake to attempt to understand the relevant propensity *solely* in terms of the abilities and motivations of individuals. As the cases of deepfakes and social bots, respectively, show, artificial intelligence may have an important role to play in the generation and distribution of fakes. Artificial intelligence can likewise be used to generate fake news reports (Fitch, 2019; Metz & Blumenthal, 2019). As others have suggested (DiResta, 2020), the process of generating and distributing fakes has in some instances already been automated, and this trend is likely to continue.^15^ To make this point stark, imagine a future in which the vast majority of online content is generated through automated systems that produce fakes with no regard for their subject matter. That this future is possible illustrates that the propensity of convincing and relevant fakes to exist in an environment is not determined solely by the properties of individuals. 

Still, there is reason to expect that the propensity of fakes to exist in an environment will be closely connected to the abilities and motivations of individuals. As anticipated by the discussion in Section 4, some actors may be motivated to create systems that will mass produce and distribute fakes, with little regard for their content, for the purposes of creating confusion. Such a strategy might be undertaken, for example, by entities aiming to reduce trust in the mainstream media, science, or other institutions. But even actors seeking to generate confusion would better fulfill their goals by distributing fakes of general interest and with emotionally laden content. It is unclear what purpose would be served, for instance, by generating fakes of persons, real or invented, engaged in banal behavior. Moreover, other actors are likely to deploy automated systems to generate fakes for more specific deceptive purposes. For this reason, there is reason to think that even those fakes created and disseminated by automated systems are most likely to involve persons and subject matters of broad interest. Thus, even if the processes of generating and distributing fakes are increasingly automated, there is reason to expect that it will continue to be the case that content involving persons and subjects matters not of broad interest is relatively likely to be authentic.

Given these considerations, the propensity view can account for certain important facts about the epistemic effects of fakes that are not explained by the ability view. Scholars concerned with the epistemic threat of deepfakes are typically concerned about the effects of such fakes on political knowledge. These concerns seem well-placed, and not purely because of the relative importance of the political domain. Rather, the importance of the political domain ensures that some parties have good pragmatic reason to fake such content—a point illustrated by the long history of misleading claims and advertisements in politics. While there are strong pragmatic reasons for which individuals can be expected to engage in fakery in the political domain, the ability to produce political fakes is no greater than the ability to produce fakes in various other domains. The ability view thus does not account for the plausible claim that fakes are especially threatening to political knowledge. In contrast, the propensity view neatly explains why fakes typically pose more of a threat to political knowledge than to, for example, knowledge of the activities of one’s friends and family. While the ability to produce fakes does not discriminate according to content, there is typically greater motivation to produce fakes concerning political events and figures. The propensity view also accounts for exceptions to this general rule. If one’s social circle includes pranksters with the technical savvy to produce convincing fakes, one’s ability to acquire knowledge concerning the activities of one’s friends and family may well be compromised. This is because of the propensity of convincing and relevant fakes to exist in one’s environment will be heightened.

A related advantage of the propensity view is that it accounts for the relative vulnerability of certain kinds of political knowledge to the epistemic threat of fakes. As I noted in Section 6, fakes plausibly pose a greater threat to the ability to acquire the knowledge that a given politician used a slur than to the acquisition of the knowledge that that politician uttered a platitude. This is easily explained by the relatively strong motivation to create fakes of politicians engaging in reputationally damaging behavior than engaging in neutral behavior. To illustrate, notice that it is more difficult to imagine a circumstances in which one would be motivated to create a fake suggesting that a politician uttered a platitude than a circumstance in which one would be motivated to create a fake suggesting that a politician uttered a slur.

The propensity view can be further motivated by appeal to the analogy with testimony. As we have seen, the mere ability of individuals within an epistemic environment to lie does not significantly compromise the attainment of warrant from testimony in that environment. Otherwise, testimonial knowledge would rarely, if ever, be possible. However, if the individuals are both able *and* motivated to lie within an epistemic environment, their propensity to lie very plausibly does undermine the attainment of testimonial knowledge in that environment. Thus, it is relatively difficult to acquire testimonial knowledge concerning used cars on a used car lot. Just as the weight of traditional testimony is compromised in environments with a heightened propensity for relevant lies to exist, I suggest that the weight of news reports, photographs, videos, and so on is compromised in environments with heightened propensities for relevant fakes to exist.

A final advantage of the propensity view over the ability view is that it accounts for the epistemic significance of emerging technologies, and especially deepfakes. As I noted above, the ability to generate fake video footage using expensive and time-consuming techniques has long existed, but without posing a serious epistemic threat to the acquisition of knowledge from video footage. The propensity view accounts for the novelty of the epistemic threat of deepfakes. While the costs of manipulating video via earlier techniques were typically prohibitive, deepfakes can be generated comparatively easily. Moreover, the trend in deepfake creation is toward increasingly user-friendly methods (Cole, 2019). But, as I began to suggest in Section 6, it is not merely the democratization of the ability to produce fakes that threatens warrant. If this were the case, then the threat to warrant in a society of saints with no temptation to fakery—but nonetheless possessing the ability to produce fakes—would resemble the threat present in our own society. Yet intuitively, this is not the case. The democratization of the ability to generate fakes matters because, as a result of this expanded ability, parties have greater all-things-considered reason to generate deepfakes than to generate fake videos using older and more costly techniques. Thus, the propensity of relevant deepfakes to exist in a typical environment is higher (or at least will be higher) than the propensity of fake videos generated through alternative means.

## The Epistemic Threat of Fakes: Limits and Defenses

Thus far in this paper, I have described some pathways by which fakes can impede knowledge acquisition and I have discussed at length the conditions under which fakes interfere with the acquisition of warrant. In this section, I discuss some limits of the epistemic threat of fakes and describe some ways in which this threat can be mitigated.

Let us begin with the limits. Keith Harris (2021) argues that the epistemic threat of deepfakes is often overstated. This is because the epistemic effects of deepfakes can be substantially mitigated by attention to the channels—including, for some examples, television channels, websites, and Twitter accounts—by which video footage is accessed. In the same way that one could be confident of the safety of trick or treating at trusted houses even if urban legends about sabotaged candy were legitimate, one can obtain knowledge from videos accessed through trusted channels even if deepfakes abound. More generally, the epistemic threat of fakes is principally a threat to the acquisition of knowledge via information spread by unfamiliar channels. Individuals may thus insulate themselves against some of the threats of fakes—especially the threat of deception—by restricting the channels by which they access information. Moreover, there is reason to think that beliefs formed by accessing such channels are warranted, despite the propensity of fakes to exist in other channels, insofar as the location of content in a trusted channel attests, over and above the content itself, to the accuracy of that content. Attention to channels may thus mitigate the threat of fakes to both truth and warrant. As suggested in Section 4, however, restricting one’s information consumption to certain channels reduces one’s opportunity for forming true beliefs.

 A second limitation on the epistemic threat of fakes is that the mere propensity of fakes to exist cannot plausibly be thought to interfere with the acquisition of knowledge. Consider an analogy. Suppose that there is, in some remote corner of the world, an eccentric billionaire whose every waking thought and deed is devoted to the construction of barn facades to decorate an elaborate network of inaccessible underground bunkers. The propensity of barn facades to exist, and indeed their actual existence in that environment, is no threat to the acquisition of knowledge of barns elsewhere. In general, the epistemic threat of fakes is plausibly restricted to the epistemic environments in which they are likely to exist. It is no easy task to discern the boundaries of epistemic environments. This difficulty has been recognized with respect to ordinary physical environments (Baumann, 2016: 56–57) and likewise arises when it comes to online epistemic environments. Despite this difficulty, the general point that the epistemic effects of fakes are restricted to their environments has concrete implications worth noting here. The existence of fakes in inaccessible environments—say, the local memory on one’s computer or private cloud storage—does not plausibly constitute an epistemic threat to internet users at large.

The preceding point naturally suggests a broad strategy for mitigating the epistemic threat of fakes. While mitigating this threat does not require preventing the creation of fakes, it does require preventing the widespread distribution of fakes into online environments. Thus, for example, measures to remove fakes from online social networks may be crucial to the abilities of users to acquire knowledge from authentic content on those platforms. This does not mean that the epistemically ideal policy regarding fakes would be to ensure that they are entirely inaccessible to internet users. There may be epistemic advantages to permitting fakes in certain online environments.^16^ For example, some philosophers argue that deepfakes may have valuable educational applications (Fallis, 2020; Westerlund, 2019). Moreover, allowing fakes in certain sections of the online environment may facilitate the identification of fakes in others by allowing the distinguishing characteristics of fakes to be studied by internet users. Relatedly, an accessible database of identified fakes might serve as a background against which other content may be compared.

This latter suggestion points toward what is perhaps the most straightforward available means for individuals to defend themselves against the epistemic threat of fakes. As I have emphasized in Sections 6 and 7, it is convincing fakes that threaten the warrant otherwise obtained from their authentic counterparts. It is also convincing fakes that are most likely to cause false beliefs. These points suggest that development of individual discriminatory abilities may go some way toward cutting off two pathways by which fakes pose an epistemic threat. Things are somewhat more complicated when it comes to the effect of fakes on the belief condition. There is some reason to think that obvious fakes pose a substantial threat to the belief condition, insofar as they make subjects aware of the threat of fakes (Rini, 2021). This point might likewise be taken to show that improving one’s discriminatory abilities with respect to fakes may have a negative effect on satisfaction of the belief condition. However, insofar as one appreciates the extent of one’s discriminatory abilities,^17^ improving those abilities may play an important role in mitigating the threat fakes pose to the belief condition. This individualistic response to the epistemic threat of fakes may be amplified and supplemented with technological techniques for labeling and restricting the accessibility of fakes.

## Concluding Remarks

I have offered an overview of some general epistemological issues surrounding online fakes. I have distinguished several varieties of fakes, some general pathways by which fakes can impede knowledge acquisition, and the conditions under which fakes interfere with warrant in particular. Finally, I have suggested some general limitations to the epistemic threat of fakes, and some ways in which this threat can be further mitigated. This general treatment of the epistemic threat of fakes should not be taken as commitment to the epistemic homogeneity of fakes. In particular, the distinct character of fake people—in the form of trolls and social bots—as both purveyors of fakes and fakes themselves calls for special attention. However, even the analysis of the distinctive character of particular kinds of fakes can be advanced by consideration of the shared features and consequences of fakes.

## Data Availability

N/A.

## Footnotes

1 This usage of “fake news” is most clearly associated with Donald Trump, but other rightwing populists in the USA have adopted the term. The term, and translations of the term, have been used elsewhere by political figures and institutions seeking to deflect criticism (Beech, 2017; Gabbatt, 2018; Isikoff, 2017; Oremus, 2022). This usage of the term also has notable historical antecedents, including *Lügenpresse* in German. This term was in use already in the nineteenth century (Beiler & Kiesler, 2018) but was most notoriously deployed for propagandistic purposes by the Nazi party (Koliska & Assmann ,^2021^).

2 For similar definitions, see Don Fallis and Kay Mathiesen (2019) and Pepp et al. (2019).

3 For a related discussion of the ideals of journalism, see Pepp and colleagues (2019: 75–76).

4 Thanks to an anonymous referee for drawing my attention to this point.

5 Thanks to an anonymous referee for recommending that I discuss higher-order evidence in this connection.

6 Fallis takes the conveyance of information to be equivalent to the conveyance of evidence.

7 Graham’s discussion is based on a variant of a widely cited case introduced by Gilbert Harman (1973). For additional discussion of this case, see Jonathan Adler (1996) and Jennifer Lackey (2008).

8 Crucially, while there is room for debate as to how narrowly to understand methods, the formation of a belief based on an authentic piece of content and the formation of a belief based on an intrinsically similar fake would involve the same method.

9 This difficulty resembles the well-known *generality problem* for reliabilism (Feldman, 1985; Goldman, 1979). As Peter Baumann (2016) argues, the generality problem is perhaps best understood as a special case of the more general *reference class problem* (Hájek, 2007; Reichenbach, 1949). In light of the difficulty of resolving these problems, it is unsurprising that it is difficult to determine a nonarbitrary principle of relevance.

10 Thanks to an anonymous referee for raising this point.

11 Fallis (2020) makes a similar point.

12 Indeed, Descartes’s claim is somewhat stronger than that central to the ability view. For Descartes, the *ineliminable possibility* that there is a deceiver capable of generating faulty perceptions is enough to generate skeptical conclusions. The ability view, by contrast, takes skeptical conclusions to be due to the *actual instantiation* of the ability to generate fakes.

13 Fallis and Kay Mathiesen (2019) develop the analogy between counterfeit currency and fake news at length.

14 One might object that the difference here is due to fact that the proposition that the politician uttered a slur has a lower prior probability than the proposition that the politician uttered a platitude. However, we can of course envision a version of the case in which it is not particularly unexpected that the politicians would use a slur. Alternatively, we could construct a version of the case in which the politician uses an unusual idiom, rather than a platitude. In either case, I submit, the knowledge that the politician used a slur remains relatively vulnerable.

15 It is worth noting that technology likely has an important role in identifying and removing fakes from online epistemic environments (Alonso et al., 2021; Khan et al., 2021; Masood et al., 2021).

16 Of course, there may be moral and other costs to this strategy as well. For example, even fakes identified as such might have a negative impact on individuals’ mental associations (Harris, 2021).

17 This point dovetails with recent work in virtue epistemology stressing the importance of knowing one’s epistemic limits (Sosa, 2015).

## References

[CR1] Adler J (1994). Testimony, trust and knowing. Journal of Philosophy..

[CR2] Adler J (1996). Transmitting knowledge. Noûs.

[CR3] Ajder, H., Patrini, G., Cavalli, F., & Cullen, L. (2019). The state of deepfakes: Landscape, threats, and impact. *Deeptrace*.

[CR4] Alonso MA, Vilares D, Gómez-Rodríguez C, Vilares J (2021). Sentiment analysis for fake news detection. Electronics.

[CR5] Bastos MT, Mercea D (2019). The Brexit botnet and user-generated hyperpartisan news. Social Science Computer Review..

[CR6] Baumann P (2008). Is knowledge safe?. American Philosophical Quarterly..

[CR7] Baumann P (2016). Epistemic Contextualism: A Defense.

[CR8] Beech, H. (2017). ‘No such thing as Rohingya’: Myanmar erases a history. *New York Times*, 2/12/217. https://www.nytimes.com/2017/12/02/world/asia/myanmar-rohingya-denial-history.html

[CR9] Beiler M, Kiesler J, Otto K, Köhler A (2018). “Lügenpresse! Lying press!” Is the press lying?. Trust in Media and Journalism Empirical Perspectives on Ethics, Norms, Impacts and Populism in Europe.

[CR10] Benkler Y, Faris R, Roberts H (2018). Network Propaganda: Manipulation, Disinformation, and Radicalization in American Politics.

[CR11] Bernecker S, Bernecker S, Flowerree AK, Grundmann T (2021). An epistemic defense of news abstinence. The Epistemology of Fake News.

[CR12] Bernecker S, Flowerree AK, Grundmann T (2021). The Epistemology of Fake News.

[CR13] Blake-Turner C (2020). Fake news, relevant alternatives, and the degradation of our epistemic environment. Inquiry.

[CR14] Bogardus T (2014). Knowledge under threat. Philosophy and Phenomenological Research.

[CR15] Broniatowski DA, Jamison AM, Qi SH, AlKulaib L, Chen T, Benton A, Quinn SC, Dredze M (2018). Weaponized health communication: Twitter bots and Russian trolls amplify the vaccine debate. American Journal of Public Health.

[CR16] Butter M, Butter M, Knight P (2020). Conspiracy theories in American history. The Routledge Handbook of Conspiracy Theories.

[CR17] Carlson, M. (2021). Skepticism and the digital information environment. *SATS – Northern European Journal of Philosophy.* 22(2), 149–167.

[CR18] Cavedon-Taylor D (2013). Photographically based knowledge. Episteme.

[CR19] Chesney B, Citron D (2019). Deep fakes: A looming challenge for privacy, democracy, and national security. California Law Review.

[CR20] Chu Z, Gianvecchio S, Wang H, Jajodia S (2012). Detecting automation of Twitter accounts: Are you a human, bot, or cyborg?. IEEE Transactions on Dependable and Secure Computing..

[CR21] Coady CAJ (1992). Testimony: A Philosophical Study.

[CR22] Cole, S. (2019). This program makes it even easier to create deepfakes. Vice News. Retrieved September 19, 2019, from https://www.vice.com/en/article/kz4amx/fsgan-program-makes-it-even-easier-to-makedeepfakes

[CR23] Comesaña J (2005). Unsafe knowledge. Synthese.

[CR24] Cox, J. (2019). Most deepfakes are used for creating non-consensual porn, not fake news. *Vice News*. Retrieved October 7, 2019, from https://www.vice.com/en/article/7x57v9/most-deepfakes-are-pornharassment-not-fake-news

[CR25] Craig E (1990). Knowledge and the State of Nature.

[CR26] Descartes, R. (1641/1995). *Meditations on First Philosophy*. In J. Cottingham, R. Stoothoff, & D. Murdoch (Eds./Trans.), *The Philosophical Writings of Descartes (Vol. II)*. Cambridge University Press.

[CR27] DiResta, R. (2020). The supply of disinformation will soon be infinite. *The Atlantic*, 20/9/2020. https://www.theatlantic.com/ideas/archive/2020/09/future-propaganda-will-be-computer-generated/616400/

[CR28] Fallis D (2020). The epistemic threat of deepfakes. Philosophy & Technology.

[CR29] Fallis, D. & Mathieson, K. (2019). Fake news is counterfeit news. Inquiry. 10.1080/0020174X.2019.1688179

[CR30] Feldman R (1985). Reliability and justification. The Monist..

[CR31] Ferrara E, Varol O, Davis C, Menczer F, Flammini A (2016). The rise of social bots. Communications of the ACM..

[CR32] Fitch, A. (2019). Readers beware: AI has learned to create fake news stories. *The Wall Street Journal*, 13/10/2019. https://www.wsj.com/articles/readers-beware-ai-has-learned-to-create-fake-news-stories-11571018640

[CR33] Fricker E, Matilal BK, Chakrabarti A (1994). Against gullibility. Knowing from Words.

[CR34] Gabbatt, A. (2018). How Trump’s ‘fake news’ gave authoritarian leaders a new weapon. *The Guardian*, 25/1/2018. https://www.theguardian.com/us-news/2018/jan/25/how-trumps-fake-news-gave-authoritarian-leaders-a-new-weapon

[CR35] Gelfert A (2018). Fake news: A definition. Informal Logic.

[CR36] Genot E, Olsson EJ, Bernecker S, Flowerree AK, Grundmann T (2021). The dissemination of scientific fake news: On the ranking of retracted articles in Google. The Epistemology of Fake News.

[CR37] Ginet C, Austin DF (1988). The fourth condition. Philosophical Analysis.

[CR38] Goldman A (1976). Discrimination and perceptual knowledge. The Journal of Philosophy.

[CR39] Goldman A, Pappas GS (1979). What is justified belief?. Justification and Knowledge: New Studies in Epistemology.

[CR40] Goldman A (1999). Knowledge in a Social World.

[CR41] González J, Torres J (2011). News for All the People: The Epic Story of Race and the American Media.

[CR42] Graham P (2000). Transferring knowledge. Noûs.

[CR43] Greco J (2010). Achieving Knowledge: A Virtue-Theoretic Account of Epistemic Normativity.

[CR44] Grimm SR (2006). Is understanding a species of knowledge. British Journal for the Philosophy of Science.

[CR45] Grundmann T (2020). Fake news: The case for a purely consumer-oriented explication. Inquiry.

[CR46] Habgood-Coote J (2019). Stop talking about fake news!. Inquiry.

[CR47] Hájek A (2007). The reference class problem is your problem too. Synthese.

[CR48] Hardwig J (1985). Epistemic dependence. Journal of Philosophy.

[CR49] Harman G (1973). Thought.

[CR50] Harris KR (2021). Video on demand: What deepfakes do and how they harm. Synthese.

[CR51] Howard, P. N. & Kollanyi, B. (2016). Bots, #StrongerIn, and #Brexit: Computational propaganda during the UK-EU Referendum. *SSRN*.

[CR52] Hughes HC, Waismel-Manor I (2020). The Macedonian fake news industry and the 2016 US election. PS: Political Science & Politics.

[CR53] Hume, D. (1748/1999). *An enquiry concerning human understanding*. In T. L. Beauchamp (ed.). New York: Oxford University Press.

[CR54] Isikoff, M. (2017). Defiant Assad tells Yahoo News torture report is ‘fake news’. *Yahoo! News*, 10/2/2017. https://www.yahoo.com/news/exclusive-defiant-assad-tells-yahoo-news-torture-report-is-fake-news-100042667.html?soc_src=social-sh&soc_trk=tw

[CR55] Jaster R, Lanius D (2018). What is fake news?. Versus.

[CR56] Kelp C (2018). Inquiry, knowledge and understanding. Synthese.

[CR57] Kerner C, Risse M (2021). Beyond porn and discreditation: Epistemic promises and perils of deepfake technology in digital lifeworlds. Moral Philosophy and Politics..

[CR58] Khan, J. Y., Khondaker, M. T. I., Afroz, S., Uddin, G., & Iqbal, A. (2021). A benchmark study on machine learning methods for fake news detection. *Machine Learning and Applications*, *4*(15), 1–12. 10.1016/j.mlwa.2021.100032

[CR59] Koliska M, Assmann K (2021). Lügenpresse: The lying press and German journalists’ responses to a stigma. Journalism.

[CR60] Lackey J (2008). Learning from Words: Testimony as a Source of Knowledge.

[CR61] Levy N (2017). The bad news about fake news. Social Epistemology Review and Reply Collective.

[CR62] Linvell DL, Warren PL (2020). Troll factories: Manufactured specialized disinformation on Twitter. Political Communication..

[CR63] Masood, M., Nawaz, M., Malik, K.M., Javed, A., & Irtaza, A. (2021). Deepfakes generation and detection: State-of-the-art open challenges countermeasures and way forward. *CoRR*, abs/2103.00484, 2021, https://arxiv.org/abs/2103.00484

[CR64] Metz, C. & Blumenthal, S. (2019). How A.I. could be weaponized to spread disinformation. *The New York Times*, 7/6/2019. https://www.nytimes.com/interactive/2019/06/07/technology/ai-text-disinformation.html

[CR65] Novaes CD, de Ridder J, Bernecker S, Flowerree AK, Grundmann T (2021). Is fake news old news?. The Epistemology of Fake News.

[CR66] Nozick R (1981). Philosophical Explanations.

[CR67] Öhman C (2020). Introducing the pervert’s dilemma: A contribution to the critique of Deepfake Pornography. Ethics and Information Technology.

[CR68] Oremus, W. (2022). In Putin’s Russia, ‘fake news’ now means real news. *The Washington Post*, 11/3/2022. https://www.washingtonpost.com/technology/2022/03/11/russia-fake-news-law-misinformation/

[CR69] Pagán VE (2008). Toward a model of conspiracy theory for ancient Rome. New German Critique..

[CR70] Pagán VE, Butter M, Knight P (2020). Conspiracy theories in the Roman Empire. The Routledge Handbook of Conspiracy Theories.

[CR71] Paris, B. & Donovan, J. (2019). Deepfakes and cheap fakes: The manipulation of audio and visual evidence. *Data & Society Research Institute*. https://datasociety.net/output/deepfakes-and-cheap-fakes/

[CR72] Paul, C. & Matthews, M. (2016). The Russian “firehose of falsehood” propaganda model: Why it might work and options to counter it. *RAND Corporation*. https://www.rand.org/pubs/perspectives/PE198.html

[CR73] Pepp J, Michaelson E, Sterken RK (2019). What’s new about fake news?. Journa of Ethics and Social Philosophy..

[CR74] Pomerantsev, P. (2014). Russia and the menace of unreality. *The Atlantic*. https://www.theatlantic.com/international/archive/2014/09/russia-putin-revolutionizing-information-warfare/379880/

[CR75] Pomerantsev P (2019). This is Not Propaganda: Adventures in the War Against Reality.

[CR76] Pritchard D (2009). Safety-based epistemology: Whither now?. Journal of Philosophical Research..

[CR77] Putnam H (1981). Reason, Truth and History.

[CR78] Reichenbach H (1949). The Theory of Probability.

[CR79] Reid, T. (1983). *Inquiry and Essays*. In Beanblossom, R. E. & Lehrer, K. (eds.). Indianapolis: Hackett.

[CR80] Rini R (2017). Fake news and partisan epistemology. Kennedy Institute of Ethics Journal.

[CR81] Rini R (2020). Deepfakes and the epistemic backstop. Philosophers’ Imprint..

[CR82] Rini R, Edenberg E, Hannon M (2021). Weaponized skepticism: An analysis of social media deception as applied political epistemology. Political Epistemology.

[CR83] Samuels, B. (2018). Russian man arrested after speaking about work at 'troll farm'. *The Hill*. https://thehill.com/policy/cybersecurity/374701-russian-man-arrested-after-speaking-about-work-at-troll-farm

[CR84] Shorey S, Howard PN (2016). Automation, big data and politics: A research review. International Journal of Communication.

[CR85] Sosa E (1999). How to defeat opposition to Moore. Noûs.

[CR86] Sosa E (2015). Judgment and Agency.

[CR87] Stengel, R. (2020). Domestic disinformation is a greater menace than foreign disinformation. *Time Magazine*. https://time.com/5860215/domestic-disinformation-growing-menace-america/

[CR88] Stroud B (1984). The Significance of Philosophical Skepticism.

[CR89] Swaine, J. (2018). Twitter admits far more Russian bots posted on election than it had disclosed. *The Guardian.* https://www.theguardian.com/technology/2018/jan/19/twitter-admits-far-more-russian-bots-posted-on-election-than-it-had-disclosed

[CR90] Teter M (2020). Blood Libel: On the Trail of an Antisemitic Myth.

[CR91] Uscinski J, Parent J (2014). American Conspiracy Theories.

[CR92] Vogel J (1990). Cartesian skepticism and inference to the best explanation. The Journal of Philosophy..

[CR93] Westerlund M (2019). The emergence of deepfake technology: A review. Technology Innovation Management Review..

[CR94] Williamson T (2000). Knowledge and Its Limits.

[CR95] Yablokov I, Butter M, Knight P (2020). Conspiracy theories in Putin’s Russia: The case of the ‘New World Order’. The Routledge Handbook of Conspiracy Theories.

[CR96] Young G (2021). Fictional Immorality and Immoral Fiction.

[CR97] Zannetou, S., Caulfield, T., De Cristofaro, E., Sirivianos, M., Stringhini, G., & Blackburn, J. (2019). Disinformation warfare: Understanding state-sponsored trolls on Twitter and their influence on the web. *Companion Proceedings of the 2019 World Wide Web Conference, ACM*. 218–226.

